# Enhancing Permeability: Unraveling the Potential of Microporous Organic Polymers in Mixed Matrix Membranes

**DOI:** 10.1021/acsapm.4c01379

**Published:** 2024-07-22

**Authors:** Alba Torres, Cenit Soto, Francisco Javier Carmona, María Teresa Simorte, Inmaculada Sanz, Raúl Muñoz, Laura Palacio, Pedro Prádanos, Antonio Hernández, Alberto Tena

**Affiliations:** † Surfaces and Porous Materials (SMAP), Associated Research Unit to CSIC, Universidad de Valladolid, Facultad de Ciencias, Paseo Belén 7, Valladolid E-47011, Spain; ‡ Institute of Sustainable Processes (ISP), Dr. Mergelina S/n, Valladolid 47011, Spain; § FCC Medio Ambiente, Avenida Camino de Santiago 40, Edificio 2 - Planta 2, Madrid 2850, Spain

**Keywords:** gas separation, mixed matrix membranes, permeability, selectivity, modeling of dual-phase permeability, *F*-factor

## Abstract

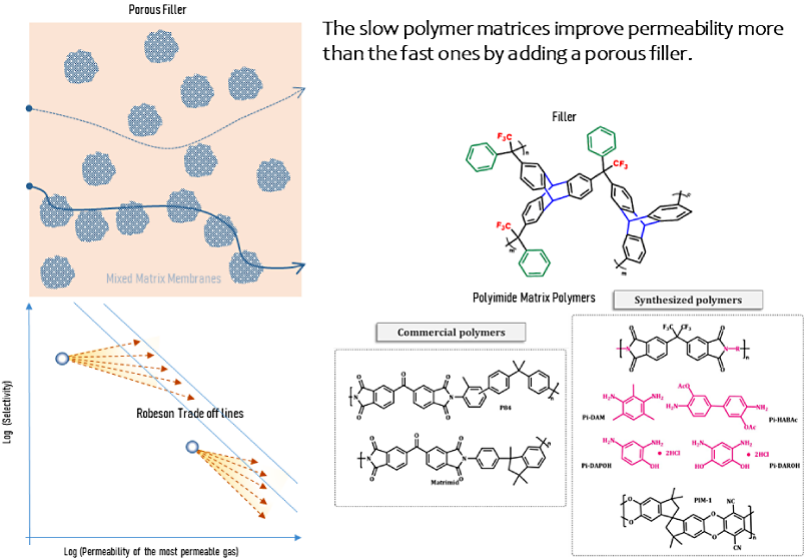

Mixed matrix membranes (MMMs) were formed by using seven polymeric matrices with a wide range of permeabilities. All of the polymeric matrices have been polyimides, namely: P84, Pi-DAPOH, Pi-DAROH, Matrimid, Pi-HABAc, PI-DAM, and PIM-1 in the order of increasing O_2_ permeability. A fixed (10%) concentration of a microporous organic polymer (TFAP-Trp), formed by the combination of trifluoroacetophenone and triptycene, was added as a porous filler. The material properties as well as their separation performances for multiple pure gases, specifically the permeabilities of He, N_2_, O_2_, CH_4_, and CO_2_, were measured. The correlation between the relative increase in permeability in MMMs and that of the matrix polymeric membrane has been quantitatively analyzed. This study proves that the increased permeability of MMMs is largely linked to the contribution of the high permeability of the filler. The addition of the TFAP-Trp porous filler proves to be especially beneficial for matrices with low to moderate permeabilities, significantly enhancing the matrix permeability overall. The fitted relationship is approximately linear in accordance with the existing models to predict permeability in dual-phase systems for low proportions of the dispersed phase. An extrapolation allows the evaluation of the permeability of the pure microporous organic polymer, which agrees with the previous values described by the group for different filler contents and in other polymeric matrices. In all cases, the selectivity remains approximately constant while the permeability increases. The addition of TFAP-Trp to all the polymeric matrices led to a moderate improvement of the MMM separation performances, mainly centered on their permeabilities.

## 1 Introduction

Membrane technology has been a widely developed tool for gas purification since the 70s due to its promising properties such as energy efficiency, low manufacturing cost, and simple-continuous operation. Membranes have demonstrated their great capacity in contemporary gas separation applications, for example, biogas sweetening, oxygen enrichment, or hydrogen purification.^1^ An extensive range of materials have been evaluated based on their gas separation performances, which are usually compared with Robeson’s plot. This plot represents the ideal selectivity versus the permeability of the most permeable gas in each pair of gases. One of the drawbacks that membranes must face is the trade-off between permeability and selectivity^2^ that compromises the performance of polymeric membranes.^3^ Some strategies have been studied to overcome this disadvantage including polymer structure tailoring,^4,5^ polymer blending,^6−8^ chain cross-linking,^9−11^ and mixed matrix membranes.^12^

Mixed matrix membranes, MMMs, are based on a homogeneous phase known as *matrix* where a solid with specific characteristics, known as *filler*, is dispersed. Out of all possibilities, mixed matrix membranes have given impressive results by enhancing permeability without decreasing significantly or even increasing the selectivity of the precursor matrix polymer.^13−16^ Obviously, one of the research strategies concerning MMMs is to optimize filler–matrix interfacial binding. In the numerous possibilities of fillers, they can be divided into two main groups: inorganic fillers such as metal–organic frameworks (MOFs), zeolites, carbon nanotubes (CNTs), or graphene nanosheets that allow easy control of pore sizes but exhibit poor compatibility with polymeric matrices;^3^ and porous organic frameworks (POFs),^16^ such as crystalline covalent–organic frameworks (COFs),^17^ porous organic cages (POCs),^18^ porous aromatic frameworks (PAFs),^19^ or porous organic polymers (POPs), have demonstrated to be an interesting option with better compatibility with organic polymeric matrices.^20,21^

POPs have undergone rapid development in recent years due to their demonstrated high performance in gas separation.^13,22^ They were commonly synthesized by the Yamamoto coupling reaction, but the use of bromine-derivative components increases the price and synthesis steps.^23,24^ Therefore, economical synthesis routes may be considered such as the Friedel–Crafts or Scholl reactions.^25^ Another limitation in the use of porous fillers is the stability of the pore. In this sense, the material conformation and integration in the polymer matrix are fundamental. A key strategy is the use of highly rigid or conformationally hindered aromatic monomers, bonded directly or via high thermal and chemical resistance groups, which do not provide conformational mobility to avoid network collapse.^26^ Following this strategy, Lopez-Iglesias et al. synthesized a set of porous polymer networks (PPNs) combining two ketones with electron-withdrawing groups and two trifunctional arenes via a hydroxyalkylation reaction under super acidic conditions.^27^ The amorphous networks obtained are highly microporous materials, showing elevated surface areas (>500 m^2^/g), with good CO_2_ capture properties and excellent chemical and thermal stability. These PPNs can be considered microporous organic polymers according to their nanometric pore sizes.^28^ Different PPNs with linear polyimides and polyamides in different loading percentages and their thermally rearranged (TR) conformation have been tested for ideal gas separation such as CO_2_/CH_4_, CO_2_/N_2_, C_2_H_4_/C_2_H_6_, and C_3_H_6_/C_3_H_8_.^12,29−32^ These studies concluded that the loading of these components contributed to increasing the permeability values without interfering significantly with selectivity. This enhancement is usually attributed to the internal porosity of the filler, the affinity of functional groups within the structure of the filler, or its capacity of adsorption.^20,31−33^

In this study, it will be investigated whether the increment of permeability of the MMM as compared with those of the pure polymeric matrix can be explained in terms of the permeability of the filler. By the study of MMMs containing a highly promising filler (trifluoroacetophenone–triptycene) in different concentrations within 6FCl-APAF, tBTpCl-APAF, and tBTmCl-APAF polyamides and their β-TR-polybezoxazoles,^34,35^ it was possible to extrapolate the permeability for a pure PPN membrane. This pure PPN-based material still presents important challenges concerning its processability, which prevents its testing as a membrane because it is almost insoluble, making it impossible to form a film. This avoids blending with the polymeric matrix and requires it to be added in the solid state. The intrinsic microporosity present in these types of compounds usually leads to materials presenting elevated permeability values. This, together with the size separation ability of the pores, results in membranes that would surpass the present Robeson’s limit for gases with sufficient differences in size such as the separation of H_2_/CH_4_.^36^ Thus, this study will correlate the increment of permeability due to the hyperhigh permeability of the filler with the separation performance of the polymer matrix. For this purpose, an ensemble of polymeric matrices with great variability inseparation performances, consisting of a set of polyimides with a wide range of permeabilities (shown in Figure 7), and a polymer with intrinsic high porosity were selected. In order to analyze the effect of the polymeric matrix, a fixed percentage, 10 wt %, of trifluoroacetophenone–triptycene was employed as the content of the porous filler. The chosen polymeric matrices have included well-described low-range permeability polymers such as P84 and Matrimid; intermediate-range permeable polymers such as Pi-HABAc, Pi-DAPOH, and Pi-DAROH; and polymers with high-range permeability such as Pi-DAM and PIM-1. All of these are described in the next section.

## 2 Materials

### 2.1 Reagents

P84 was purchased from HP Polymer GmbH (Lenzing, Austria). Matrimid was purchased from Huntsman Advanced Materials GmbH (Bergkamen, Germany). Pi-HABAc, Pi-DAM, Pi-DAPOH, and Pi-DAROH were obtained from the polycondensation reaction of 4,4′-(hexafluoroisopropylidene)diphthalic anhydride, 6FDA, purchased from Fluorochem (Glossop, UK), and the corresponding diamino monomers: 3,3′-dihydroxybenzidine, HAB, from Apollo Scientific (Manchester, UK); 2,4,6-trimethyl-1,3-benzenediamine, DAM, from Apollo Scientific (Manchester, UK); 2,4-diaminophenol dihydrochloride, DAP·2HCl, from Merck-Sigma-Aldrich (Missouri, USA); and 4,6-diaminoresorcinol dichloride, DAR·2HCl, from BLD Pharmatech GmbH (Kaiserslautern, Germany). All monomers were used without further purification. The polymer with intrinsic microporosity, PIM-1, was synthesized by the reaction of 5,5′,6,6′-tetrahydroxy-3,3,3′,3′-tetramethyl-1,1′-spirobisindane, TTSBI, and 2,3,5,6-tetrafluoro-1,4-dicyanobenzene, TFTPN, in the presence of anhydrous potassium carbonate, K_2_CO_3_, all purchased from Merck-Sigma-Aldrich.

PPN-TFAP-Trp was obtained by the reaction of triptycene, Trp, from Fluorochem (Glossop, UK) with 2,2,2-trifluoroacetophenone, TFAP, in the presence of trifluoromethanesulfonic acid, TFSA, both purchased from Apollo Scientific (Manchester, UK). Anhydrous 1-methyl-2-pyrrolidinone (NMP), dimethylacetamide (DMAc), anhydrous pyridine (Py), anhydrous dimethylformamide (DMF), acetic anhydride, and anhydrous chloroform were purchased from Merck-Sigma-Aldrich. *o*-Xylene was obtained from VWR International (Pennsylvania, USA) and ethanol was obtained from Quimilid (Valladolid, Spain). All solvents were used as purchased.

## 3 Experimental Section

### 3.1 Characterization Techniques

Proton nuclear magnetic resonance spectra (^1^H NMR) were obtained using a Bruker Advance instrument (Bruker, Billerica, MA, USA) working at 400 MHz. Carbon solid-state magnetic resonance spectra (^13^C_solid_-NMR) were recorded at 100.6 MHz with a solid-state Bruker Avance 400 (Mannheim, Germany), equipped with a superconducting wide magnet, magic angle spinning, and cross-polarization.

Attenuated total reflectance-Fourier transform infrared (ATR-FTIR) spectrometry was performed using a PerkinElmer Spectrum One FT-IR (PerkinElmer, Waltham, MA, USA) coupled with a universal diamond-tipped sampling module from 4000 to 400 cm^–1^ with 4 cm^–1^ of resolution.

Size exclusion chromatography was used to determine the number and weight-average molecular weights, *M*_n_ and *M*_w_ respectively, and the polydispersity index using a Waters permeation chromatograph equipped with a Waters 2414 refractive index detector (Waters, Milford, MA, USA). Polystyrene standards (Polymer Laboratories, Church Stretton, UK) were used for the calibration in all cases. Samples with good solubility in DMF were analyzed using a set of Styragel HR3 and HR5 Waters columns. A solution of *N*,*N*-dimethylformamide (DMF) with 0.1% LiBr was used as the mobile phase. Samples that were not soluble in DMF were analyzed by using a set of HR4, HR1, and HR0.5 Waters columns with HPLC-grade THF as the solvent.

Thermogravimetric analysis (TGA) was carried out using a TA Instruments-Waters Corp. A TGA 550 thermogravimetric analyzer (TA Instruments, Milford, USA) with a Hi-Resolution ramp of 10 °C/min from 30 to 800 °C in the presence of an N_2_ (99.999%) purge gas flux of 40 mL/min.

Differential scanning calorimetry (DSC) was carried out using a TA Instruments modulated DSC-25 analyzer (TA Instruments, Milford, MA, USA) to establish glass transition temperatures (*T*_g_). DSC thermograms were obtained by a double heating procedure. The first heating rate was from 20 to 400 °C, then cooling down at 20 °C/min until 40 °C, and finally, a heating rate of 20 °C/min up to 400 °C under an N_2_ atmosphere using a 10 mg sample in gastight aluminum containers. TRIOS software (TA Instruments, Milford, MA, USA) was used for the computational treatment of data for TGA and DSC results as the onset point of the degradation slope and with the inflection method of the slope after the second heating cycle, respectively.

### 3.2 Polymer Synthesis

The synthesized polyimides were made by a stoichiometric two-step polycondensation reaction as described in earlier works.^37^ In a previously dried three-necked flask equipped with a mechanical stirrer and under an inert atmosphere, diamine (HAB, DAM, DAP·2HCl, and DAR·2HCl) was added and dissolved in NMP as a solvent. The use of pyridine (10 mmol/1 mmol diamine) was necessary when using DAP·2HCl and DAR·2HCl, in order to get rid of salt protection. Then, the mixture was cooled with an ice bath for 15 min, and 6FDA was added. The solution was left to warm to room temperature and left overnight, obtaining a viscous transparent solution. Chemical imidization was carried out for HAB and DAM by adding 4.5 mmol of acetic anhydride and 4.5 mmol of pyridine, and stirring at room temperature for 5 h. For DAP and DAR, it is important to highlight that the chemical imidization was tested. However, the polymers imidized in this way did not reach a sufficient molecular weight. Then, azeotropic imidization was carried out for DAP- and DAR-containing polymers by adding half of the solvent volume of *o*-xylene to the solution and vigorously stirring and heating for 6 h at 180 °C. Later, the *o*-xylene was distilled from the polymer solution. Finally, for every polymer, the mixture was cooled and poured into water. Afterward, the precipitated polymer was washed five times with water and ethanol, and then dried at 150 °C for 12 h under vacuum. The synthesized polyimides have been identified as Pi-HABAc, Pi-DAM, Pi-DAPOH, and Pi-DAROH.

The polymer of intrinsic microporosity, PIM-1, was obtained through the formation of a dibenzodioxin structure as a polycondensation product previously reported.^38,39^ Under a nitrogen atmosphere, a mixture of stoichiometric amounts of TTSBI and TFTPN was dissolved in anhydrous DMF (100 mL). Then, an excess of anhydrous K_2_CO_3_ (2.5 equiv) was added and stirred at 65 °C for 72 h. Upon cooling, the mixture was poured into methanol (300 mL) to precipitate the crude polymer. The product was collected by filtration and washed with water to remove any salts. The polymer was dissolved in THF (100 mL) and reprecipitated using methanol twice. The fluorescent yellow polymer was collected by vacuum filtration and dried at 120 °C for 12 h.

**Figure 1:**
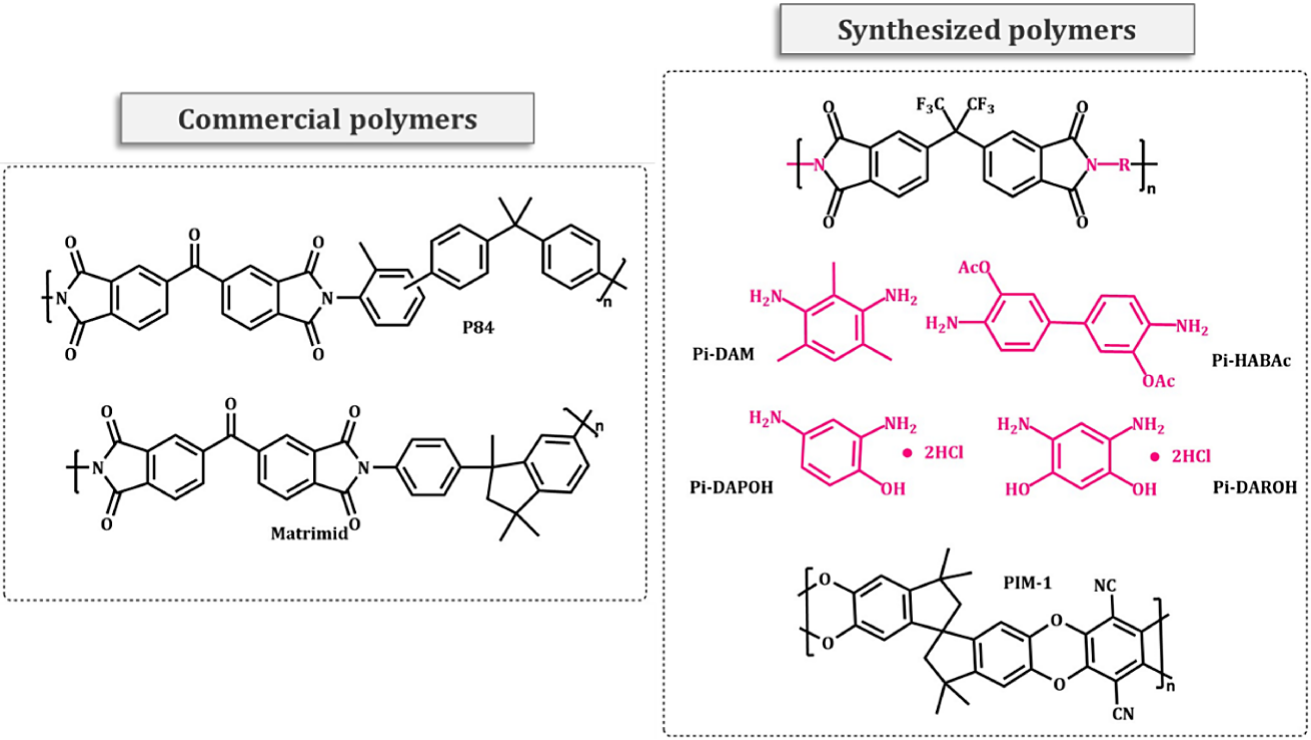
Chemical structures of synthetic and commercial polyimides.

The chemical structures of the polymers used in this work are shown in Figure 1. The structures of the synthesized polymers were confirmed by ^1^H NMR. Some properties of these polymers are as follows **Pi-HABAc**: ^1^H NMR (400 MHz, DMSO-*d*_6_) δ 8.20 (d, 2H), 7.98 (d, 2H), 7.84 (s, 2H), 7.81–7.75 (m, 4H), 7.66 (d, 2H), 2.14 (s, 6H). FTIR (film): imide ν(C =O) at 1777 and 1716 cm^–1^, imide ν(C–N) at 1368 cm^–1^. GPC analysis (DMF + 0.1% LiBr): *M*_n_: 14 862 g mol^–1^, *M*_w_: 24 103 g mol^–1^ relative to polystyrene; *M*_w_/*M*_n_ = 1.62. **Pi-DAM:**^1^H NMR (400 MHz, DMSO-*d*_6_) δ 8.21 (d, 2H), 7.99–7.92 (m, 4H), 7.35 (s, 1H), 2.17 (s, 4H), 1.95 (s, 2H). FTIR (film): imide ν(C=O) at 1788 and 1723 cm^–1^, imide ν(C–N) at 1355 cm^–1^. GPC analysis (DMF + 0.1% LiBr): *M*_n_: 17 034 g mol^–1^, *M*_w_: 25 298 g mol^–1^ relative to polystyrene; *M*_w_/*M*_n_ = 1.49. **Pi-DAPOH**: ^1^H NMR (400 MHz, DMSO-*d*_6_) δ 10.33 (s, 1H, OH), 8.20 (dd, 2H), 7.97 (d, 2H), 7.81 (dd, 2H), 7.42 (s, 1H), 7.16 (d, 2H). FTIR (film): alcohol ν(−OH) 3700–3000 cm^–1^, imide ν(C=O) at 1785 and 1714 cm^–1^, and imide ν(C–N) at 1360 cm^–1^. GPC analysis (DMF + 0.1% LiBr): *M*_n_: 36 288 g mol^–1^, *M*_w_: 89 781 g mol^–1^ relative to polystyrene; *M*_w_/*M*_n_ = 2.47. **Pi-DAROH**: ^1^H NMR (500 MHz, DMSO-*d*_6_) δ 10.20 (s, 2H), 8.19 (d, 2H), 7.99 (d, 2H), 7.84 (m, 2H), 7.24 (s, 1H), 6.67 (s, 1H). FTIR (film): alcohol ν(−OH) 3700–3000 cm^–1^, imide ν(C=O) at 1785 and 1714 cm^–1^, imide ν(C–N) at 1360 cm^–1^. GPC analysis (DMF + 0.1% LiBr): *M*_n_: 13 617 g mol^–1^, *M*_w_: 23 788 g mol^–1^ relative to polystyrene; *M*_w_/*M*_n_ = 1.75. **PIM-1:**^1^H NMR (400 MHz, CDCl_3_) 6.8 (s, 2H), 6.5 (s, 2H), 2.1–2.4 (m, 4H), 1.2–1.4 (m, 12H). FTIR (film): ν(C–H) at 2900–2800 cm^–1^, ν(−CN) at 2240 cm^–1^, and ν(C–O) at 1262 cm^–1^. GPC analysis (THF): *M*_n_: 18 750 g mol^–1^, *M*_w_: 1 16 040 g mol^–1^ relative to polystyrene; *M*_w_/*M*_n_ = 6.19.

### 3.3 Synthesis of the PPN

The microporous polymer, TFAP-Trp, was synthesized in high yields (>95%) using the methodology reported in a previous work^40^ by a hydroxyalkylation reaction. TFSA was used to get superacidic conditions so that the protonation of the ketone in TFAP may occur, followed by an electrophilic aromatic substitution on Trp. In this way, a three-necked flask with a mechanical stirrer and gas inlet and outlet was charged with TFAP (5.8 mmol), triptycene (3.9 mmol), and chloroform (9 mL). The mixture was stirred at room temperature under a nitrogen atmosphere and cooled to 0 °C, and TFSA (8.6 mL) was added dropwise during 15 min. Then, the mixture was allowed to warm to room temperature and stirred for 5 more days. The obtained solid was then poured into a water/ethanol mixture (3/1), filtered, and consecutively washed with water, acetone, and chloroform. Finally, the product was ground and dried at 150 °C for 12 h in a vacuum.

**Figure 2:**
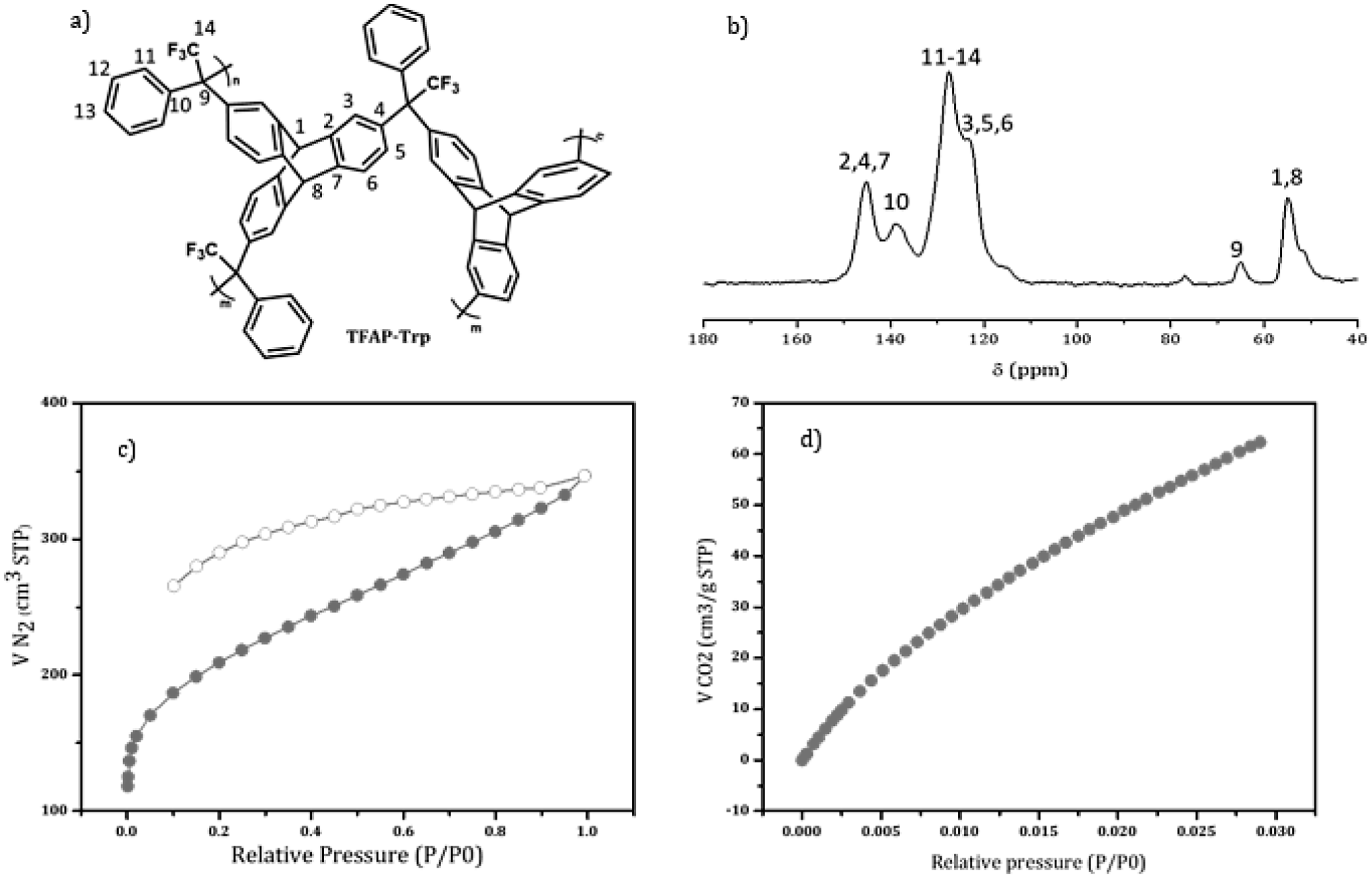
a) Chemical structure, b) ^13^C_solid_-NMR assignment, and adsorption (solid circle)–desorption (empty circle) isotherms of c) N_2_ at 77 K and d) CO_2_ (right) at 273.15 K for the TFAP-Trp molecule.

**Figure 3:**
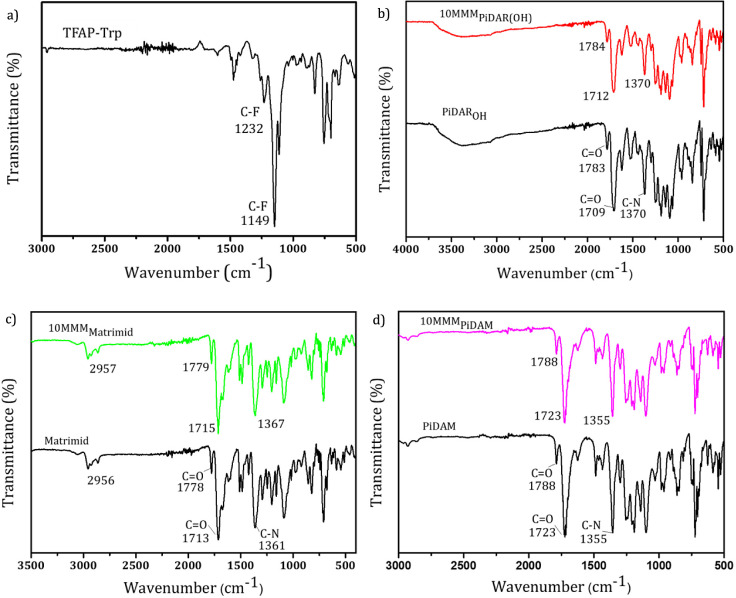
FTIR spectra of a) TFAP-Trp, b) Pi-DARPOH, c) Matrimid, and d) PiDAM pristine and MMMs.

The TFAP-Trp structure (Figure 2a) was confirmed by the ^13^C_solid_-NMR solid, as shown in Figure 2b. In the FTIR spectrum (Figure 3a), the C–F vibration signals of TFAP were identified at 1234 and 1145 cm^–1^ as reported.^27^ According to the IUPAC assignment for the adsorption isotherms of N_2_, the filler presents a microporous profile (Figure 2c). The BET specific surface area was 761.8 m^2^ g^–1^. The adsorption isotherm of CO_2_ has allowed the determination of the CO_2_ adsorbed at 273.15 K (2.8 mmol g^–1^) and total pore volume (62.3 cm^3^ g^–1^) for a ratio *P*/*P*_0_ = 0.029 (corresponding to 1 bar) (Figure 2d). The standard method to determine the pore volume and microporous size distribution from CO_2_ isotherms requires working at 273.15 K. Other higher temperatures cause less adsorption but with the same size distribution, as proved by Soto et al.^28^

### 3.4 Formation of the Mixed Matrix Membranes

Membranes (0 and 10 wt % PPN content) were formed by the solution casting method. The solvent and the drying protocol employed for each case are described in Table 1. First, a suitable amount of the polymer was dissolved, and the solution was filtered through a 3.1 μm fiberglass Symta (Symta SSL, Madrid, Spain) filter in order to eliminate any insoluble part. Concurrently, a suspension in the same solvent with a precise amount of PPN was sonicated for 20 min (40 cycles of 20 s of ultrasounds with 30% of amplitude followed by 10 s pauses) with a S250D digital sonifier equipped with a 102-C converter (Branson Ultrasonics-Emerson Electric, Brookfield, CT, USA) of 130 W to eliminate agglomerations and to ensure a good and homogeneous dispersion of the filler. Then, the suspension was added to the previously prepared polymer solution and mixed by fast magnetic stirring. Finally, the solution was cast onto a leveled glass plate and slowly dried according to the corresponding protocol, as described in Table 1. The thickness of the films obtained in this way was measured with a Dualscope MP0R from Fischer (Fischer, Waldachtal, Baden-Wurttemberg, Germany). The resulting thicknesses of the membranes both with and without the filler, without significant differences depending on the presence of the filler, are 60 ± 15 μm.

**Table 1:** Solvent and Drying for MMM Manufacture According to the Matrix Polymer

polymer	solvent	drying
Matrimid	THF	room temperature until dry and 120 °C for 12 h under vacuum.
P84, Pi-HABAc, Pi-DAPOH, Pi-DAROH, Pi-DAM	NMP	60 °C for 12 h and 100 °C for 1 h. Finally, until 300 °C under an N_2_ atmosphere.
PIM-1	THF	room temperature until dry, washed with new MeOH for 1, 2, and 24 h, and dried to 120 °C for 24 h under vacuum.

### 3.5 Gas Transport Properties

The single gas permeabilities of He, O_2_, N_2_, CH_4_, and CO_2_ were measured at 35 °C and an upstream pressure of 3 bar, which is the pressure drop commonly used for the sake of comparison. A homemade constant-volume, variable-pressure permeation system was used.

The membranes were degassed under high vacuum for 12 h to eliminate humidity and any other adsorbed gases. The absence of pinholes was determined by measuring the helium permeability coefficient at three different pressures (1, 2, and 3 bar). A scheme of the permeator operated to use the time lag procedure is shown elsewhere.^41^ According to this method, the permeability coefficients (Pi, Barrer = 10–10 (cm^3^ (STP) cm)/(cm^2^ s cmHg)), under steady-state conditions, can be obtained using eq 1:
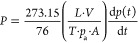
1where *V* is the downstream volume (cm^3^), *A* is the effective area (cm^2^), *L* is the thickness of the membranes (cm), *T* is the operational temperature (K), *p*_a_ is the pressure of the feed gas (bar), and  is the slope of downstream pressure versus time. The well-known time lag method is described in detail elsewhere.^41^

The ideal selectivity α_X/Y_, for two gases X and Y, was calculated as the ratio of their single gas permeabilities as follows:
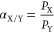
2

## 4 Results and Discussion

### 4.1 Characterization of the Mixed Matrix Membranes

FTIR analyses were performed for reference and mixed matrix membranes and, respectively, were compared to determine any matrix–filler interaction. No differences were observed between their spectra, which would indicate that the interaction between the polymeric matrix and the filler does not change the chemical structure of the materials, although it could be also due to a very homogeneous distribution of the filler, leading to very small concentrations of the filler in the areas where the FTIR spectrum was recorded, which is mainly limited to the surface of the samples. This would lead to a very weak signal of the PPN present in the MMMs and a negligible change in the background signal corresponding to the polymeric matrix. Probably, higher loading contents of the porous filler might lead to different signals. In any case, the signals associated with the filler were not detected here. The same phenomena were reported by Tariq et al. using Matrimid and loadings of up to 30% of microporous 3D Tb(BTC)(H_2_O).(DMF)_1.1_ MOF,^42^ Aguilar-Lugo et al. with Pi-HABOH, Pi-HABAc, and their corresponding TRPBO in 15–30% of microporous isatin-triptycene polymer,^29^ and Chen et al. with 1–7% of MOF-801 in PIM-1.^43^ It also confirms no clear chemical interaction between the filler and the matrix. The situation can be different in the case of rubbery polymers, as Kang et al. reported with 15% loads of ZIF-8, which can be attributed to the rubbery character of the polymeric matrix as well as to the inorganic character of the filler, leading to a modification of the IR peaks of the polymer but still without perceiving filler signals.^14^ Some results for the materials obtained in this work are shown in Figure 3. The isolated filler (Figure 3a) shows an intensive peak at around 1232 and 1149 cm^–1^ related to the vibration of the C–F bond present in TFAP-Trp. The comparison of Pi-DAROH and MMM_Pi-DAROH_ is shown in Figure 3b. As mentioned before, no clear differences are identified between both spectra, and especially, the signal related to TFAP-Trp cannot be identified.

**Figure 4:**
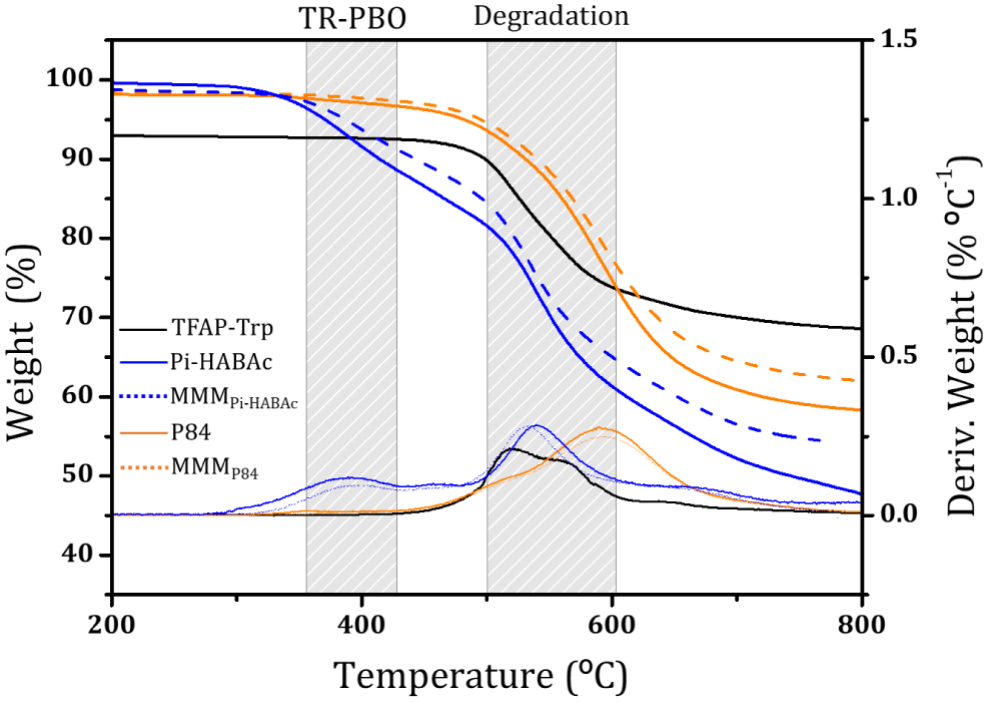
Thermograms for Pi-HABAc, P84, and their MMMs.

The thermal stabilities for the corresponding MOP, pristine polymers, and MMMs were studied by TGA. Additionally, the weight losses associated with intramolecular processes of some of the pristine polymers, and their corresponding MMMs were also identified by TGA. The thermograms for Pi-HABAc (which can be thermally rearranged) along with P84 (which cannot be thermally rearranged) are shown in Figure 4. The thermogram for Pi-HABAc shows two different steps. The first one in the range of 300–500 °C is where the thermal rearrangement of polyimide to polybenzoxazole takes place.^6^ The second one in the range of 500–600 °C corresponds to the decomposition of the polymer. This behavior is observed for all of the polyimides with the possibility to reach thermal rearrangement. The thermogram for P84 shows a single weight loss around 500 °C corresponding to the degradation of the polymer. Thermogram shows an initial weight loss (<4%) which corresponds to the release of residual solvent or absorbed water. The thermal behavior was similar for Pi-DAM and Matrimid.

Degradation temperatures, *T*_d_, and glass-transition temperatures, *T*_g_, of all membranes, and for our MOP, are shown in Table 2. The *T*_g_s for Pi-DAM and PIM-1 membranes were not detected. Due to their highly rigid structure and limitation of conformational degrees of freedom, *T*_g_ for PIM-1 has been historically difficult to be detected with DSC. However, recently, Yin et al.^44^ have discussed the possibility of measuring it by several heating cycles with fast scanning calorimetry (FSC), with very fast temperature ramps, and reported a *T*_g_ of 443 °C. The *T*_d_s from the neat membranes and the corresponding MMMs have minor discrepancies, if any. Note that TFAP-Trp has almost as high decomposition temperature as the matrix polymers, which should explain the substantial lack of effect of its incorporation on the matrix polymers when forming the corresponding MMMs.

**Table 2:** Thermal Parameters of Precursor Polymers and MMMs

polymer	*T*_g_ (°C)^a^	*T*_d_ (°C)^b^
TFAP-Trp	nd^c^	490
Matrimid	324	497
MMM_Matrimid_	328	498
P84	325	519
MMM_P84_	329	520
Pi-HABAc	278,^47^ 267^45^	513
MMM_Pi-HABAc_	328	506
Pi-DAM	397,^48^ 390,^49^ 395^50^	508
MMM_Pi-DAM_	nd^c^	508
Pi-DAP_OH_	355	524
MMM_Pi-DAPOH_	371	522
Pi-DAR_OH_	329	513
MMM_Pi-DAROH_	nd^c^	511
PIM-1	nd^c^	493
MMM_PIM-1_	nd^c^	500

a Middle point of the endothermic step.

b Degradation temperature from the maximum point in the last weight loss determined by TGA.

c Glass transition temperature was not detected.

The MMM_Pi-DAPOH_ is the one with the highest increase of the glass transition temperature as compared to the corresponding matrix membrane without differing from the decomposition temperature. This Pi-DAPOH polymer shows less stiffness due to the skeletal structure, which might lead to a better interaction with the porous filler. The results on the *T*_g_ for Pi-HABAc are quite unexpected because, although Pi-HABAc is a well-described polymer,^45−47^ the *T*_g_ for the pristine membrane has been impossible to determine. The increase between the reported neat membrane *T*_g_ and that for the corresponding MMM can be attributed to the conversion of the acetate group to hydroxyl when heating at high temperatures for long time as studied in the literature.^45^ Therefore, the explanation can be related to the drying protocol, which has favored the presence of a large proportion of hydroxyl groups. For this reason, the high *T*_g_ of the MMM_Pi-HABAc_ (328 °C) is much closer to the Pi-HABOH one (300 °C^46^ and 356 °C^47^) than to that for the Pi-HABAc (278^47^ and 267 °C^45^).

### 4.2 Gas Transport Properties

**Figure 5:**
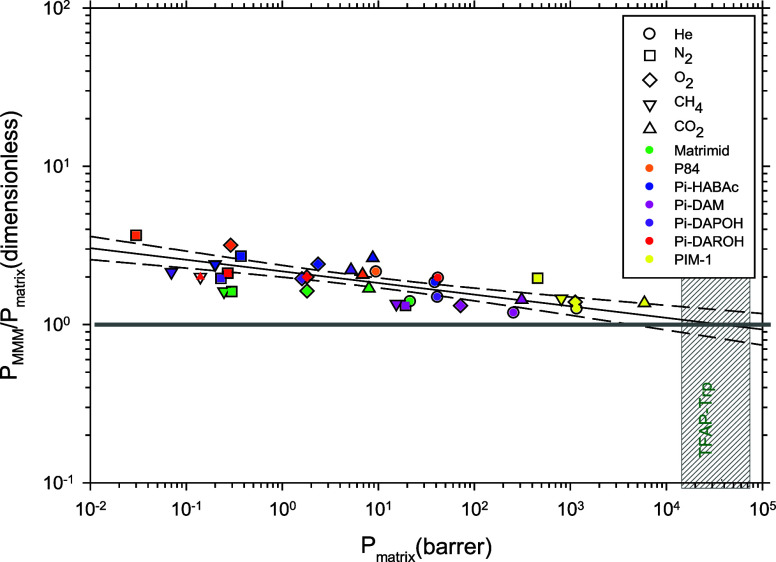
P_MMM_ /P_matrix_ as a function of P_matrix_. The extrapolation to P_MMM_ = P_matrix_ is also shown. This extrapolation should correspond to pure filler permeability. The vertical striped band corresponds to the value extrapolated by Soto et al.^34,35^ Dashed and point straights correspond to the fitting 95% confidence and prediction intervals, respectively, while the solid line corresponds to the linear fitting.

The permeability of the MMMs per unit of the pure polymeric matrix permeability versus the matrix permeability for all of the gases and membranes tested is shown in Figure 5. For P84, the permeability of CH_4_ was not measured because the gas transport performance was too low and close to or lower than the order of the error of the pressure detector.

In Figure 5, the degree of improvement of the permeability of all the gases tested for the materials is shown. This degree of improvement of the permeability is shown by the ratio between the permeability of the MMMs and that of the pristine polymer matrix. These ratios show that the permeability increases more for the low permeability polymers. This is represented by a larger permeability ratio, which should be 1 when there is no impact on the performance. On the contrary, polymers in the high permeability range, such as Pi-DAM and PIM-1, show much lower increase of permeability. In any case, all the polymers have shown an increase in permeability after adding 10% of the TFAP-Trp filler.

Note that for the high permeability polymers, the filler is expected to have less impact, while for low permeability polymers, the change in permeability can be more significant. In some cases, there is extra permeability over the fitting line, which would mean that an extra free volume has been created. This extra free volume could be due to the presence of intervoids, which might massively improve the permeability while having a much lower effect on selectivity.^28,37,51^ In other cases, permeability is lower than expected, which could be attributed to a certain shielding of the filler due to a more compact packing of the polymer chains around the porous filler or even to a partial blocking of the porosity of the filler. In any case, low permeability polymers show a massive increase of permeability, almost 250% for P84 for example. Medium permeability polymers, such as Pi-DAROH or Pi-DAPOH, show a great increase in the permeability, around 100%. While fast polymers show a still interesting, moderate improvement of around 35–40%. All these facts show that, in principle, the permeability of TFAP-Trp is higher than any of the polymeric matrices^52^ tested in this work, in accordance with the results shown in Figure 5. It is important to keep in mind that the improvement to which we are referring corresponds purely to permeability.

Note that there is a common tendency that can be fitted to a decreasing power for all of the membranes and gases. This trend covers a broad range of permeabilities. Accordingly, as displayed in Figure 5:
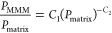
3

Or:
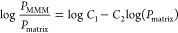
4

And:
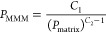
5

Here, both constants are
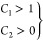
6

Of course, *C*_1_ should be necessarily positive as far as permeabilities are always positive but it is also *C*_1_ > 1 in our case. Note that eq 3 means that the addition of small amounts of the filler is especially beneficial when the polymeric matrix has low to moderate permeabilities when *C*_2_ > 0 which is the case here. It is also relevant to take into account that the slope of the solid line straight in Figure 5 is quite low, specifically eq 5 gives (*C*_2_ = 0.07):
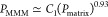
7

This obviously means that MMMs always have higher permeabilities than the corresponding matrix polymer membrane. Consequently:
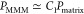
8

This is a result predicted by all the models^53,54^ to forecast the permeability of dual-phase systems for low enough proportions of the disperse phase, which is the case here.

Some models exclusively intended to contain small amounts of the filler within the continuous matrix. Among these low load models, we can cite those due to Bruggeman, Bötcher, and De Loor^55^ that would give different functions of the volume fraction of the filler ϕ:
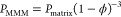
9
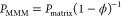
10
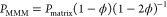
11

The corresponding multiplicative constants would be 1.37, 1.11, and 1.13, respectively, while from the fitting data, we get *C*_1_ = 1.9 ± 0.8 in best accordance with the Brugeman’s model although in accordance, within the error range, with the three models.

**Figure 6:**
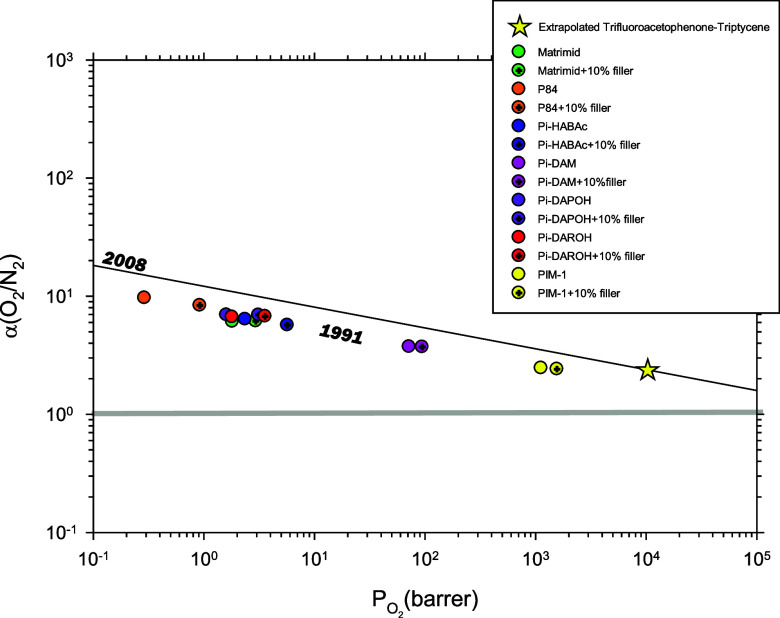
Selectivity versus permeability for the O_2_/N_2_ pair of gases. The corresponding values for the pristine microporous organic polymer as extrapolated for these two gases in a plot like that in Figure 5 for both gases separately. The corresponding trade-off lines are also shown.

Note, as well that the continuous straight line in Figure 5 can be extrapolated for high permeabilities to cross the line defined by *P*_MMM_ = *P*_matrix_. This intercept should correspond to the permeability of the pure microporous organic polymer and here it is equal (within the error range) to the interpolation for the same PPN, but with increasing the filler contents in other membranes, and for the H_2_/CH_4_ gases, as given by Soto et al.^35^ When this is done for different gases, we get the selectivity versus permeability for the microporous organic polymer. This leads to Robeson plots, such as the one for O_2_/N_2_ that is shown as an example in Figure 6.

**Figure 7:**
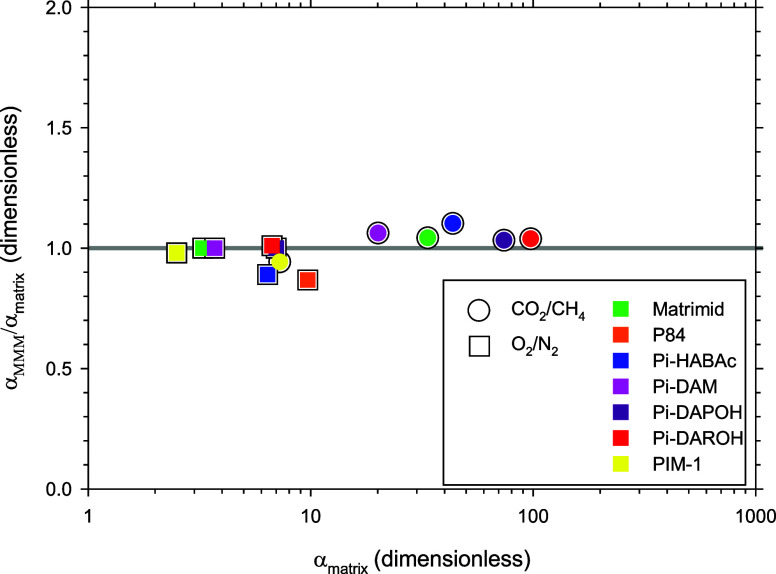
α _MMM_ /α _matrix_ as a function of α_matrix._ Note that all MMMs contain 10% TFAP-Trp filler.

Note that, the Robeson plot shown here suggests that in most cases, higher proportions of the filler would lead, if ideal interactions and filler distribution could be maintained, to a better permeability versus selectivity balance because the 1991 Robeson trade-off line can be approached or even exceeded. In Figure 7, the corresponding selectivities for the gas pairs CO_2_/CH_4_ and O_2_/N_2_ are shown for the studied membranes. There it can be seen that there is no definite tendency. However, the selectivity of MMMs is mostly determined by the polymeric matrix as far as  for all the membranes, and pairs of gases shown. Therefore, it seems clear that the inclusion of these TFAP-Trp porous fillers does not affect the selectivity of the materials. In fact, only in the case of the materials with a larger percentage of improvement of permeability, such as P84® and Pi-HABAc, the selectivities of the MMMs are slightly lower than the selectivities of the polymeric matrices (Figure 7). For the CO_2_/CH_4_ pair, all the polymers maintained or showed a very small increase in their selectivity, indicating that the contribution of TFAF-Trp is mainly affecting the creation of extra volume. Good compatibility between both phases has been generally obtained independently of the range of permeability of the polymeric matrices. Clearly, the significant increase in the permeability for all the cases, together with maintaining the sieving ability of the materials, leads to an introduction of a competitive change in the materials.

A thorough and powerful procedure to evaluate the true effect of the filler consists of applying a criterion called *F*-index, which has been described in the literature.^56,57^ The *F*-index is defined in terms of permeability and selectivity as follows:
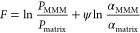
12

Here, permeabilities refer to the most permeable gas in the pair with selectivities α_MMM_ and α_matrix_ and ψ is a coefficient, which is equal to the absolute value of the slope of the upper bound to be used as a reference in a Robeson’s plot. The *F*-index would express the gas separation quality of MMMs and classify it within the ranges of <0, 0–1.5, 1.5–4, 4–8, and >8 that represent the insufficient, moderate, competent, exemplary, and ideal qualities, respectively.^56,57^

In our case, if we assume that α_MMM_/α_matrix_ ≃ thus:
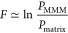
13

Then, according to eq 8
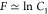
14

**Figure 8:**
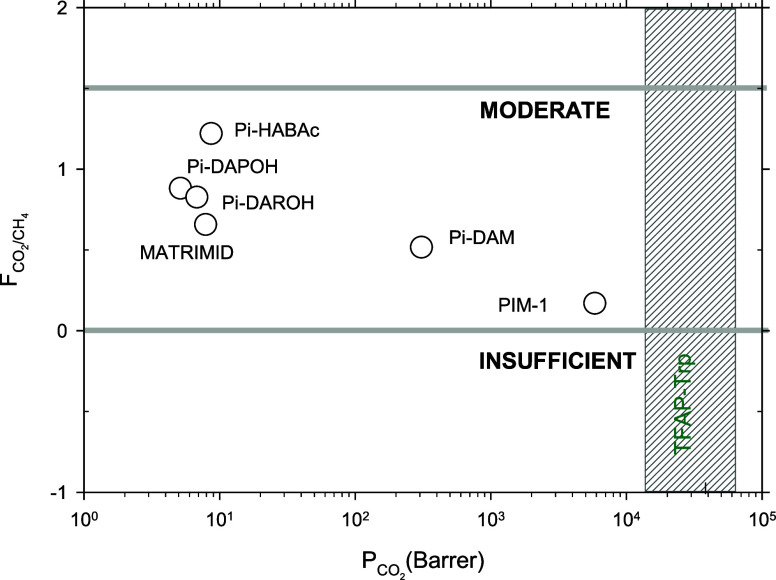
*F*-index for the MMM for CO_2_/CH_4_ separation as a function of the CO_2_ permeability of the pristine polymer matrix membranes. The extrapolation of the fitted straight to  is also shown. This should correspond to pure filler CO_2_ permeability. The vertical striped band corresponds to the value extrapolated by Soto et al.^34,35^

Because we are dealing with many polymers and gases and actually 0.9<α_MMM_/α_matrix_ < 1.1, the corresponding *F*-index would be an average one. Finally, looking at Figure 7 and eq 4, and attending to the prediction intervals in Figure 7, we get 0.7< *F* < 1.6with a central value of *F* = 1.1. This would mean that our filler should be mostly moderate, attending to its qualities in MMMs for all the polymers and gas pairs. If the actual individual *F*-indexes are evaluated by taking into account that α_MMM_/α_matrix_ ≠ 1 but using their actual values and taking the Robeson’s 2008 upper bound^36^ as a reference, the results for the CO_2_/CH_4_ pairs are shown, as an example, in Figure 8. There, it is seen that Pi-HABAc gives the best *F*-index and the highly permeable polymers, such as PIM-1, give the worst index for the CO_2_/CH_4_ separation.

Note that the dotted line shown in Figure 8 can be extrapolated to  which according to eq 9 should correspond to *P*_MMM_ = *P*_matrix_. This intercept corresponds to the high permeability extrapolation shown in Figures 5 and 6. Additionally, it is observed, once again, that there is a tendency to obtain lower results, attending to the *F*-index for highly permeable polymeric matrices. Slow polymers would thus give better *F*-index MMMs. As discussed before, Pi-HABAc somehow separates from the tendency due to nonideal interactions between the filler and the polymeric matrix. Since the dominant factor is the ratio between permeabilities, as shown in eq 10 and Figure 5, the evolution of the *F*-index follows the same tendency. Polymers showing a larger improvement, or slow-medium permeability polymers, show a higher *F*-index, while the polymers with a lower degree of improvement, or fast permeability polymers, show a lower *F*-index.

## 5 Conclusions

This study suggests that the increased permeability of the mixed matrix membrane may be in effect attributed to the high permeability of the filler material. The relationship between the increased permeability and the permeability of the filler material has been quantitatively studied. This has been accomplished by studying MMMs formed by using seven polymeric matrices with a wide range of different permeabilities as matrices filled with a fixed (10%) content of trifluoroacetophenone–triptycene.

The addition of microporous organic fillers is especially beneficial, increasing permeability, when the polymeric matrix has low to moderate permeabilities. However, in all cases, the addition of the filler led to an improvement of the permeability independent of the initial permeability of the polymeric matrix. In particular, it has been seen that *P*_MMM_ ≃ *C*_1_*P*_matrix_. This outcome aligns with the predictions made by the models proposed in the literature to predict the permeability of dual-phase systems, particularly when considering low proportions of the dispersed phase, which is the scenario in this case, and ideal dispersions.

The extrapolation of such dependence of *P*_MMM_/*P*_matrix_ on *P*_matrix_ for *P*_MMM_/*P*_matrix_ = 1 gives an intercept in fair agreement with the value obtained previously by us for the same compound but with increasing contents of the same microporous organic polymer within other polymeric matrices.

Of course, a sole increase in permeability does not automatically lead to a better membrane as far as it is the permeability–selectivity compromise that is more relevant. In our case, it has been shown that the selectivity is generally maintained with the independence of the polymeric matrix. While for polymers presenting low to medium permeabilities, a slight decrease was detected. For materials presenting high permeability values, a modest increase was found. Finally, considering permeability and selectivity, all of the membranes gave moderately better results for all of the tested gases after adding the filler.
